# Towards Effective, Sustainable Solution for Hospital Wastewater Treatment to Cope with the Post-Pandemic Era

**DOI:** 10.3390/ijerph20042854

**Published:** 2023-02-06

**Authors:** Ang Liu, Yaqian Zhao, Yamei Cai, Peiying Kang, Yulong Huang, Min Li, Anran Yang

**Affiliations:** 1 State Key Laboratory of Eco-Hydraulics in Northwest Arid Region, Xi’an University of Technology, Xi’an 710048, China; 2 Department of Municipal and Environmental Engineering, School of Water Resources and Hydroelectric Engineering, Xi’an University of Technology, Xi’an 710048, China

**Keywords:** hospital wastewater, constructed wetlands, SARS-CoV-2, MBR, Fenton oxidation

## Abstract

Severe acute respiratory syndrome coronavirus-2 (SARS-CoV-2) has spread across the globe since the end of 2019, posing significant challenges for global medical facilities and human health. Treatment of hospital wastewater is vitally important under this special circumstance. However, there is a shortage of studies on the sustainable wastewater treatment processes utilized by hospitals. Based on a review of the research trends regarding hospital wastewater treatment in the past three years of the COVID-19 outbreak, this review overviews the existing hospital wastewater treatment processes. It is clear that activated sludge processes (ASPs) and the use of membrane bioreactors (MBRs) are the major and effective treatment techniques applied to hospital wastewater. Advanced technology (such as Fenton oxidation, electrocoagulation, etc.) has also achieved good results, but the use of such technology remains small scale for the moment and poses some side effects, including increased cost. More interestingly, this review reveals the increased use of constructed wetlands (CWs) as an eco-solution for hospital wastewater treatment and then focuses in slightly more detail on examining the roles and mechanisms of CWs’ components with respect to purifying hospital wastewater and compares their removal efficiency with other treatment processes. It is believed that a multi-stage CW system with various intensifications or CWs incorporated with other treatment processes constitute an effective, sustainable solution for hospital wastewater treatment in order to cope with the post-pandemic era.

## 1. Introduction

By the end of 2021, 1,044,000 medical facilities, including 36,000 hospitals, were established in China [1]. Hospitals offer patients medical exams, therapy, nursing, and consultations, while a hospital’s treatment department, laboratories, wards, and living facilities for administrative employees all generate wastewater [2]. Due to its varied sources, hospital wastewater contains a high organic load, heavy metals, bacteria, and viruses [3]. During the era of epidemics, medical resources have been constrained, resulting in a substantial quantity of hospital wastewater [2]. Hence, the safe treatment of hospital wastewater is particularly important.

**Figure 1:**
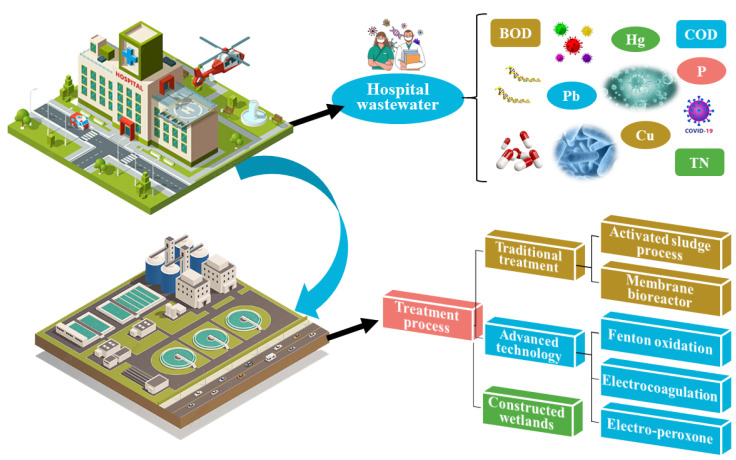
A schematic summary of hospital wastewater and its treatment processes.

During the evolution of wastewater treatment technology, the activated sludge process was the first to emerge. It was the treatment process most commonly employed in wastewater treatment plants (WWTPs) [4]. The activated sludge process (ASP) effectively removes the majority of macromolecular pollutants but is ineffective against bacteria and viruses. Thus, membrane bioreactors (MBRs) were employed to aid sludge–water separation. An MBR’s built-in filter membrane has a small pore size and can filter the majority of pollutants in hospital wastewater [5]. Advanced techniques, such as Fenton oxidation, electrocoagulation, and the electro-peroxone process, can be used to successfully remove organic matter and drugs. However, such techniques are rarely employed in actual engineering due to technical problems and expenses [6]. Constructed wetlands (CWs) constitute a sustainable treatment process with low cost, simple operation, and landscape value [7]. In recent years, there have been a number of reports regarding the treatment of hospital wastewater by CWs systems. A brief summary of the features of hospital wastewater and its treatment processes is illustrated in Figure 1.

In the past three years since the outbreak of COVID-19, numerous studies and review articles have been published on hospital wastewater treatment-related topics. These articles reflect the current demand for hospital wastewater treatment and the urgency of the scenario. Ajala et al. [8] analyzed the concentration, fate, and environmental impact of selected dugs (Carbamazepine, Ofloxacin, Clofibric acid, Ciprofloxacin, and Norfloxacin) in hospital wastewater. Majumder et al. [9] examined the efficacy of various hospital wastewater treatment processes with respect to removing antibiotics, resistance genes, and resistant microorganisms, as well as SARS-CoV-2 inhibition measures. Once in the sewage system, drugs may travel through various pathways, showing great environmental stability and persistence, or volatilization, as well as chemical or biological degradation [8]. Drugs containing both alkaline and acidic functional groups such as ciprofloxacin and ceftazidime exhibit more complex behaviors in sewer networks and WWTPs [8]. Khan et al. [10] overviewed the overall impact of hospital wastewater on WWTPs from its entry, the removal of various emerging pollutants, and environmental risks in the pretreatment, secondary, and tertiary treatment stages. In the past three years, more attention has been paid to the fate of anti-COVID-19 drugs in hospital wastewater throughout the entire aquatic environment. Recently, Cappelli et al. [11] assessed the effect of anti-COVID-19 drugs on aquatic ecosystems. More studies have begun to focus on these issues, and it is believed that hospital wastewater is facing new challenges and should be carefully investigated in order to cope with the post-pandemic era or novel future pandemics.

Through reviewing the relevant literature and the extraction of the main ideas of these articles, we were able to identify the most important hospital wastewater issues, including the challenges and reasonable solutions, from an effective, eco-friendly, and sustainable basis. Based on the published literature and by bridging the knowledge gap, this review begins with the status of the hospital wastewater treatment profile and identifies the new challenges under the current situation. The focus then shifts to the various processes used and that have emerged for hospital wastewater treatment. Thereafter, the review clarifies the decontamination principle of each component of the CWs and their performance in terms of hospital wastewater treatment efficiency. The ecological value and engineering issues of CWs are discussed via comparison with other treatment processes. Accordingly, the multi-stage CW system can be identified as an efficient and sustainable treatment process for hospital wastewater treatment while coping with the post-pandemic era.

## 2. Research Trends in Hospital Wastewater Treatment since the COVID-19 Outbreak

**Figure 2:**
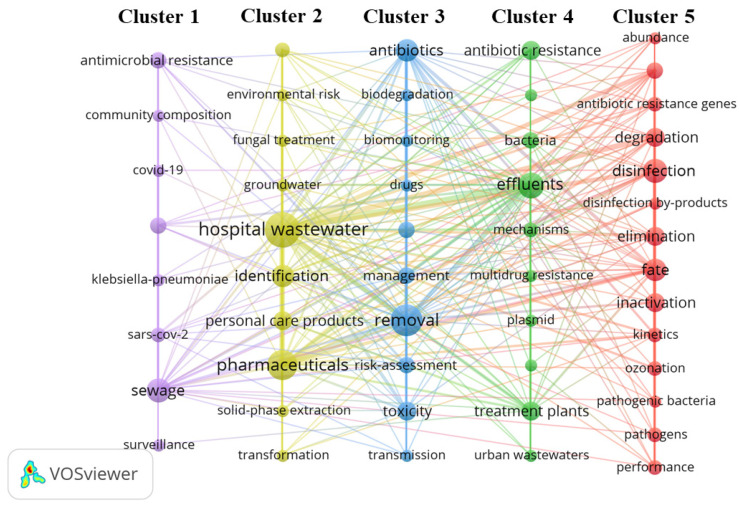
Keyword network clustering map for hospital wastewater treatment (The diameter of the circle is positively correlated with the frequency of keywords).

To determine the research status and trends in hospital wastewater treatment over the past three years, we searched the Web of Science database using the following search terms: “hospital wastewater” OR “hospital wastewaters” OR “hospital effluent” OR “hospital sewage” OR “waste water from hospital”) AND (“treatment” OR “treated” OR “treating” OR “management” OR “detection” OR “disinfection” OR “surveillance” OR “monitoring”. Research from 1 January 2020 to 31 December 2022 was searched; the retrieved content included the title, abstract, the author’s keywords, and keywords plus. Thus, 445 papers were obtained, including review articles. A screening procedure was then performed using the software VOS viewer (version 1.6.18), and the steps included the deletion of unrelated and similar keywords, followed by the drawing of a keyword network figure and its modification using Pajek (version 5.16). There are five clusters in the figure, each representing a different research direction (Figure 2). Cluster 1 includes the monitoring of SARS-CoV-2 in sewage, the bacterial community composition in sewage, etc. Cluster 2 consists of pharmaceuticals, personal care products (PPCPs), and water transfer. Antibiotics, their removal, and risk assessment are included in Cluster 3. Antibiotic resistance and bacteria in water and sewage treatment plants constitute Cluster 4. Cluster 5 consists primarily of disinfection (ozone) and disinfection by-products, a profusion of antibiotic resistance genes, and active pathogens.

Undoubtedly, the monitoring of SARS-CoV-2 in hospital wastewater and the disinfection of treated effluent were the main focuses, without regard to the treatment processed employed, of research over the past three years.

## 3. Challenges of Hospital Wastewater Treatment

A multitude of causes including drug residues, waste iodine contrast agents, viral transmission, and the excessive growth of germs, especially during the epidemic era, contribute to the complex composition of hospital wastewater [12]. Hospital wastewater contains a considerably higher concentration of drug residues (antibiotics, β-receptor blockers, NSAIDs, analgesics, etc.) than municipal wastewater [13]. Human pathogens such as adenovirus, hepatitis A virus, and tuberculosis virus are common in hospital wastewater [14]. These viruses may be hiding in the feces, urine, or vomit of infected individuals, entering the city’s sewage system via hospital wastewater [15]. Furthermore, a substantial proportion of severe acute respiratory syndrome coronavirus-2 (SARS-CoV-2) may be present in hospital wastewater throughout the entire water circulation system, thus necessitating further water treatment.

Indeed, most countries and organizations have established legislation or guidelines for treating and discharging hospital wastewater. In China, the State Environmental Protection Administration issued the “Water Pollutant Discharge Standard for Medical Institutions” in 2006, which regulates the concentration or content of COD, BOD, phosphorus (P), halogen, and fecal coliform bacteria in hospital wastewater [16]. In the United States, the “Clean Water Act” was adopted [17], while the “Special Waste Regulations” were applied in the United Kingdom [18] and the “Biomedical Waste Management and Disposal Rules” were used in India [19]. Very few nations or organizations have enacted pharmaceutical regulations, such as the “List of Toxic and Harmful Pollutants” compiled by the Environmental Protection Agency of the United States, which includes erythromycin and five synthetic hormones, and the “Watch List” formulated by the European Union, including anti-inflammatory drugs “Diclofenac” and three antibiotics: “erythromycin, clarithromycin and azithromycin” [20]. There have been several reports on the discovery of antibiotics, resistance genes, and resistant bacteria in WWTPs or aquatic environments, yet institutional restrictions are minimal [2,21].

The anti-COVID-19 drugs recommended for use during the epidemic are listed in Table 1. The variety of drugs used in hospitals is increasing, and this increase was particularly notable during the early stages of the pandemic. Due to the lack of clarity regarding SARS-CoV-2, drug abuse is common. It is understandable that saving human lives is more important than protecting the environment, so hospital wastewater may contain more drug residues in the pandemic period [11]. From April to December 2020, Cappelli et al. [11] conducted a monthly surveillance campaign at three WWTPs where temporal trends in certain anti-COVID-19 drugs were positively correlated with COVID-19 cases and deaths. The WWTPs received effluent from a hospital that specialized in treating patients with COVID-19, so the concentrations of hydroxychloroquine, azithromycin, and ciprofloxacin were among the highest. However, in the post-epidemic era, hospital treatment protocols have matured, and further consideration must be given to the environmental impacts, the resistance genes introduced by the excessive use of antibiotics and other drugs, and the concentrations of trace micro-pollutants, and the treatment of all these concerns should all be gradually improved in future regulations [22]. However, with the gradual liberalization of virus control policies in some countries, the number of people infected with SARS-CoV-2 has increased, hospital admissions have increased dramatically, and the amount of hospital wastewater generated has become enormous.

It seems that the adherence to inadequate systems including tight legislations or guidelines with which to consider the lessons learned from the last three years of the pandemic may result in an increased discharge of hazardous substances from hospital wastewater, endangering both humans and the environment, especially in the ensuing post-pandemic era. These issues represent the real challenges for this kind of wastewater treatment.

## 4. Hospital Wastewater Treatment Processes

The information and summary of the traditional treatments, advanced technology, and CWs regarding hospital wastewater from the literature are listed in Table 2. Each of the techniques is introduced from a technical perspective as well as with respect to their pros and cons for the purpose of identifying the best solution for hospital wastewater treatment.

### 4.1. Traditional Treatment

The ASP and MBRs have a long history of technological advancement [4,35]. The two techniques have similar mechanisms of biological treatment. After mixing activated sludge with wastewater, a large volume of bacteria can biodegrade various pollutants via bio-respiration. This is followed by sludge–water separation to generate the effluent [4]. The ASP has played a key role in hospital wastewater treatment in the past, but its large volume of excess sludge production during treatment and final disposal makes it tedious and costly with respect to sustainable applications. Undoubtedly, MBRs enhance the separation of sludge and water. An MBR separates sludge from water using a filter membrane. The treatment is effective, and the occupancy area is small. The literature on traditional hospital wastewater treatment processes is presented in Table 2.

The combined process associated with the ASP can be used to eliminate pharmaceuticals and other contaminants, and such combinations include ASP followed by the use of a biofilter [25] or dosing activated carbon [36]. Mir-tutusaus et al. [24] reported an average removal rate of 83% for 22 pharmaceuticals when the ASP was combined with H_2_O_2_. In some studies, the removal rate of quinolones such as norfloxacin, ofloxacin, and ciprofloxacin by an MBR exceeded 90% [26,27,37,38,39]. An MBR’s filter membrane is often a microfiltration or ultrafiltration membrane. If the MBR device does not effectively treat bacteria or viruses, the addition of a nanofiltration membrane (1–2 nm) or reverse osmosis membrane (0.1–0.7 nm) is necessary. Most bacteria and viruses (including SARS-CoV-2) have a diameter larger than these parameters, and thus the filtering ability is sufficient to eliminate these pathogenic microorganisms [5,20].

As time passes, the filtration performance of the filter membrane will deteriorate, resulting in membrane fouling. At this point, the cleaning or replacement of the membrane is required [40]; otherwise, filtration efficiency will be reduced. Furthermore, models based on mathematics, artificial neural networks, random forests, and other technologies can forecast membrane fouling. Emphasis should be placed on the implementation of these technologies [41].

### 4.2. Advanced Technologies

The literature on the treatment of hospital wastewater by advanced technology is shown in Table 2. Fenton oxidation, photocatalysis, electrocoagulation, and the electro-peroxone process are effective for the removal of both organic matter and drugs from wastewater.

Fenton oxidation is the oxidation of contaminants by hydroxyl radicals (•OH) generated by Fenton reagents (Fe^2+^ and H_2_O_2_), and the process is suited to the treatment of industrial wastewater and landfill leachate [42]. According to a scientific paper, •OH attacks the molecular structure of trace contaminants in three distinct ways: (1) H-abstraction, (2) single-electron transfer, and (3) electrophilic addition (hydroxylation) [43]. In recent years, Fenton oxidation has been increasingly used, but its use had mainly been small scale [28]. The electro-peroxone process and Fenton oxidation both rely on the great oxidation ability of •OH to eliminate contaminants from wastewater. The electro-peroxone process utilizes electricity as a catalyst to enhance ozone oxidation, resulting in a more effective treatment than ozone and significantly reduced battery usage [44,45]. In many cases, the highest contaminant-treatment efficiency can be achieved by adjusting just a few parameters (ozone flow rate, initial solution pH, applied current, etc.) [46]. Catalytic wet oxidation is appropriate for treating wastewater with a high organic load (approximately 10 to 100 g/L COD) [47]. At 150 °C, Segura et al. [28] utilized catalytic wet oxidation to eliminate 98% of COD and 90% of total pharmaceuticals from hospital wastewater.

In addition, the combination of multiple advanced technologies can improve treatment outcomes. Kashani et al. [6] added an iron electrode (as a sacrificial electrode) to treat hospital wastewater based on an electro-peroxone device and treated it under optimal conditions (initial PH = 3, ozone 33.1 mg/L, applied current 0.18 A) for 40 min. Resultantly, ciprofloxacin was eliminated, while the TOC removal rate surpassed 70%. Indeed, multiple kinds of electro-peroxone, electro-Fenton, ozone oxidation, and electrocoagulation processes coexist in this system, of which each possesses a remarkably strong oxidizing capacity. In addition, this combination confers a disinfecting action that can eliminate the majority of organic matter, pharmaceuticals, and pathogens in hospital wastewater [31,48,49]. This is a promising technique for the treatment of hospital wastewater. Fenton oxidation requires H_2_O_2_ and higher temperatures, whereas photocatalysis, electrocoagulation, and the electro-peroxone process require a great amount of electrical energy [6]. These advanced technologies can effectively treat hospital wastewater, but due to the expenses and technical difficulties involved in their use, they have not become popular and have not been implemented in large-scale operations. Furthermore, the combined process may have negative impacts that reduce removal effectiveness [50]. Segura et al. [28] enhanced Fenton oxidation at 70 °C to remove 70% and 50% of COD and TOC in hospital wastewater, respectively. The removal rate of 78 kinds of drugs reached 99.8% in a photocatalytic coupling Fenton oxidation technique, but it was discovered that photocatalysis could hinder Fenton oxidation’s capacity to remove COD and TOC. The latter COD removal rate declined to 30% under the same conditions, while the TOC removal rate was only 5%. Currently, the mechanism of action is still unclear.

### 4.3. Constructed Wetlands

#### 4.3.1. Mechanism of Pollutant Removal by Components of CWs

**Figure 3:**
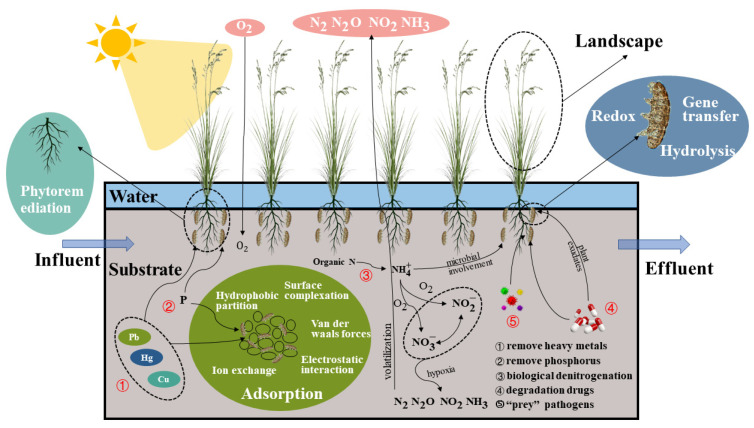
Mechanism of pollutant removal by components of CWs.

A CW is a green, sustainable wastewater treatment technology with striking features such as ecologically restorative functions, low operational costs, and low energy consumption, and it has been widely used globally for various wastewater treatments, especially in recent years [2,7,32,33]. In CWs, the substrate, plants, and microbial community all collaborate to eliminate contaminants from wastewater. Figure 3 depicts the mechanism of contaminant elimination by each component.

##### Substrates

For many years, gravel, sand, and soil have been common substrates for CWs [51]. These substrates provide a habitat for microbes. Through van der Waals interactions, surface complexation, hydrophobic partitioning, electrostatic interactions, and ion exchange, the substrate adsorbs contaminants [52].

A CW’s substrate plays a key role with respect to pollutant removal, and thus seeking alternative/novel substrates represents important CWs research and development. It is necessary to choose a substrate that exerts a strong removal effect towards antibiotics, resistance genes, and other pollutants in order to treat hospital wastewater. Zeolites have an exceptional capacity to eliminate antibiotics and resistance genes [53]. Lightly expanded clay aggregate (LECA) is a novel substrate that contains alkaline oxides and carbonates. It has good P removal activity, conductivity, and high mechanical strength; can provide improved plant rooting and biofilm growth support [54]; and through its use pharmaceutically active chemicals (carbamazepine, diclofenac, and ibuprofen) and nutrients are efficiently removed from hospital effluent [55]. A consensus has been reached regarding the characteristics of the sustainable development of CW systems; one such characteristic is the availability of a broad selection of substrates—based on the concept of waste utilization—with which to select some of the so-called waste materials. Alum sludge generated in water treatment plants will ultimately be landfilled or burned. However, alum sludge, as a potential substrate of a CWs due to its outstanding absorption performance, is of tremendous importance from a waste utilization perspective [56]. In addition, the coupling of alum sludge-based CWs with the ASP offers a greater capacity for P adsorption and can serve as a habitat for microbes, thereby increasing the biomass of the aeration tank, microbial activity, ammonia nitrogen load, and hydraulic load [57]. In addition to alum sludge, other waste such as broken bricks and coal ash can also be utilized as a substrate, and these types of waste are more successful with respect to eliminating antibiotics and P.

With regard to CWs, substrate clogging is a thorny problem. Biological and abiotic factors contribute to the clogging of substrates. The excessive growth of biofilm and extracellular polymers on the substrate constitute the biological reason, whereas the accumulation of organic matter and suspended matter constitute the abiotic cause [58]. There are several technical methods used to alleviate this condition. Aside from the replacement of the clogging substrate, some technical measures, including the change of the operation mode, such as the anti-sized arrangement of the substrate [59]; the use of composite CWs [60]; intermittent operation [61]; and tidal flow CWs [62,63], are usually employed. Additionally, the occurrence of substrate clogging can be predicted, for which a certain mathematical model needs to be established [64].

##### Plants

Common reeds, *Scirpus validus,* rushes, cattails, etc., are the typical plant species present in CWs [65]. These plants consist of two parts: one is the stem and leaves above ground, which can be considered to be a landscape contributor, and the other is the rhizosphere below ground, which offers a living environment for bacteria and can eliminate antibiotics and pathogens [66]. For example, *Scirpus validus* can eliminate paracetamol [34].

Dires et al. [67] compared the nitrogen and P removal capabilities of planted (sugar cane) and non-planted CWs and discovered that planted wetlands had a greater capacity to eliminate nitrogen and P, which was probably due to the stimulation of the plant rhizosphere to produce more microbes. Through phytoremediation [68], plants remove contaminants such as antibiotics, heavy metals, and pathogens. This involves plant adsorbents, root exudation, and microbial degradation. The influence of a plant adsorbent is negligible in comparison to that of root exudates and microbial degradation [69,70,71]. The roots accumulate the most pollutants among plant tissues [72], but the pollutants may migrate upward; thus, the potential risk of plant harvesting (antibiotic enrichment) should be seriously considered [73,74].

##### Microbes

Different plant rhizospheres have varying densities of microbes. Chen et al. [71] utilized denaturing gradient gel electrophoresis to measure the microbial density in the rhizospheres of various plant species. The ranking of microbe density is as follows: *Canna indica*, *Cyperus flabelliformis*, *Hymenocallis littoralis*, and *Iris. Tectorum*, in descending order. Even in the rhizosphere of the same plant, the density of microbes may vary, which may be correlated with the availability of nutrients, pH, temperature, and the humidity of the plant’s living environment [75].

Microbes destroy pollutants via redox reactions, gene transfer, hydrolysis, etc. [76]. Under aerobic and anaerobic conditions, ammonification, nitrification, and denitrification bacteria can eliminate nitrogen from wastewater [77]. This alternative aerobic and anaerobic environment is afforded by CWs’ distinctive structure. Antibiotics can also be degraded by microbes, such as ammonia-oxidizing microorganisms, which can eliminate antibiotics [78]. *Curvularia* can effectively eradicate erythromycin [79]. Microbes in CW environments can “prey” on pathogens. Wand et al. [80] created CWs by planting a mixture of rushes and reeds and employing coarse sand as a substrate. The primary elimination process for *E. coli* is the ability of *leelovibrio* and protozoa to “prey” on the bacteria. In the investigation conducted by Proakis et al. [81], a similar “prey” mechanism was also observed in rotifers. There are few reports on the microbial degradation of pathogens in CWs, and thus further research is required.

##### The Interaction among Substrates, Plants, and Microbes

When treating wastewater, CWs rely on the synergy of the substrate, plants, and microbes, wherein the substrate is the most important part, as it provides a habitat for bacteria and plants and plays a crucial role in the process of eliminating pollutants.

By producing particular molecules that mediate the link between roots and microbes, plant roots “choose” the microbial community that is beneficial to their survival [82], which affects the microbe density and diversity in the roots [71]. The term “choose” may refer to the habitat in which the plant thrives. If the plant does not acquire the necessary microbial community, its growth will be stunted, and it may even die [83]. Nitrogen-fixing bacteria, such as *Bacillus* and *Paenibacillus* species, enable plants to uptake nitrogen [77]. Certain root system bacteria affect the uptake of orthophosphate by plants [84]. Iron is a critical trace element for chlorophyll synthesis. Certain volatile organic compounds (VOCs) generated by rhizobia “signal” to plants to increase iron absorption by acidifying plant roots and boosting iron reductase activity [85]. Nonetheless, plants may also pose a hazard to the survival of microbes. For instance, the alkaloids released by *Nuphar lutea* inhibit the action of microbes, and even the phenolic compounds generated by certain plants are toxic to microbes [86].

#### 4.3.2. Why Constructed Wetlands Are Being Chosen

**Figure 4:**
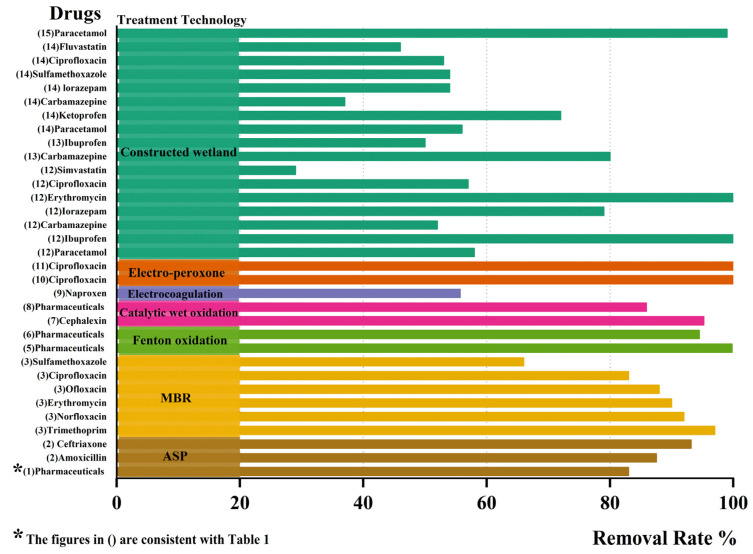
Various treatment processes’ rates of drug removal from hospital wastewater.

Table 2 shows the literature on the treatment of hospital wastewater by CWs, while Table 3 summarizes the removal rates of conventional water quality indicators by various treatment technologies. Compared to municipal sewage, the removal rate of hospital wastewater treatment has decreased, which may be due to the presence of numerous harmful substances in hospital wastewater (Table 3). However, there is a greater focus on the removal of drugs from hospital wastewater, particularly through the use of advanced technology. In addition, this study examines the rate at which the medication is eliminated from all treatment processes. Some studies represented individual medicines and showed each drug’s name, while others represented the average rate of drug elimination. These drugs are listed as a type of “pharmaceutical” in the figure below, and its vertical coordinates represent the removal rate. As depicted in Figure 4, the removal efficacy of the advanced technique is generally greater. The average removal rate of medicines by Fenton oxidation has surpassed 94%, and ciprofloxacin has been eliminated by the electro-peroxone process. An MBR has a powerful ability to eliminate drugs, with the majority exceeding 80%. The degree of sulfamethoxazole removal by an MBR is suboptimal, with a 66% removal rate. The removal efficiency of drugs by CWs is about 50% or less, with more details shown in Figure 4. Some studies have reported that some drugs such as tramadol, sulfamethoxazole, carbamazepine, and fluoxetine offer negative removal levels in CWs. The cause of this may be the fact that the effluent concentration is higher than the influent concentration due to the infiltration and transpiration-induced wastewater concentration. It is also likely that the drug (Carbamazepine) is metabolized and excreted in the form of glucuronide or other conjugates, which are transformed back into the parent compound by an enzymatic reaction [87,88,89,90].

In recent years, researchers have made efforts to maximize the full potential of CWs to treat hospital wastewater. These include developing intensified CWs (such as continuous aeration CWs, the use of novel substrates in CWs, embedding microbial fuel cells into CWs, etc.), and the incorporation/combination of other treatment processes into/with CWs (such as tube-settlers, Fenton oxidation, etc.). Lutterbeck et al. [91] treated hospital laundry wastewater with microbial fuel cells and CWs, and the removal rates of COD, BOD_5_, and TN were 79.8%, 78.6%, and 81.6%, respectively. Using graphite and granular carbon electrodes, the maximum open-circuit voltages of microbial fuel cells are 148 mV and 268 mV, respectively. The coupling system has potential from an economic and environmental development perspective [92]. Khan et al. [32,93] combined CWs with tube-settlers to treat hospital wastewater. The results demonstrated a significant increase in the organic matter and pharmaceutical removal rates. When hospital wastewater with a high drug load is treated in CWs, a quantity of H_2_O_2_ is generated, which places some stress on the plants that is primarily caused by paracetamol [34]. To alleviate this stress, plants will release catalase to degrade H_2_O_2_. Furthermore, when Fe^2+^ is present in the system, the Fenton reaction will produce •OH with significant oxidizing power, hence enhancing the potential ability of hospital wastewater to remove pharmaceuticals [43,94]. Aeration may affect the redox potential of wetland ecosystems, which, in turn, affects the degradation of drugs by microbes [32]. Auvinen et al. [87] developed a CW and applied continuous aeration to treat hospital wastewater; consequently, the removal rates of metformin and valsartan were dramatically boosted (99 ± 1% for metformin versus 68 ± 32% and 99 ± 1% for valsartan versus 17 ± 19%, respectively).

Aside from the enhanced treatment performance of integrated CWs, a CW is a low-carbon technology [95]. Chen et al. [96] estimated the greenhouse gas emissions of CWs and typical conventional sewage treatment plants during their construction and operation phases and discovered that the carbon intensity of CWs was considerably lower than that of conventional wastewater treatment systems. Ecologically speaking, the promotion of CWs is good over the long run. CWs not only perform effectively in terms of wastewater treatment but also add a substantial amount of organic matter to the soil after the decomposition of plant debris. Additionally, the plants’ biomass can be used to produce energy [97,98]. CWs play a positive role in sustaining biomass, managing water storage, and refilling groundwater [99,100]. Additionally, CWs incorporate beautiful plants offer a certain emotional value to hospital patients, thereby rendering them more acceptable than other conventional techniques [65]. Ecological restoration projects employing CWs are being implemented globally [100,101,102]. Undoubtedly, the use of CWs combined with other treatment processes is recommended and can play a greater role in hospital wastewater treatment.

**Table 3:** Various treatment processes’ rates of conventional water quality indexes concerning drug removal from hospital wastewater.

	CWs	ASP	MBR	Fenton Oxidation	Catalytic Wet Oxidation	Electrocoagulation	Electro-Peroxone
COD	(1) 79.8–94%	(2) 89.5–97.1%	(3) 89–99%	(4) 30–98%	(5) 98%	(6) 75.5–98.4%	(7) 90–94.3%
BOD	(8) 78.6–96%					(9) 59.2–97.9%	
TN	(10) 65.6–81.6%		(11) 52–65%				
TP	(12) 51.7–58.7%	(13) 61.1%	(14) 27.9%				

Reference: (1). [91,93,103]. (2). [25,104,105,106]. (3). [27,37,38,39,107]. (4). [28,108]. (5). [28]. (6). [30,109,110]. (7). [31,46]. (8). [67,91,92,103,111]. (9). [30,109]. (10). [91,92,112]. (11). [27,39,107]. (12). [112,113]. (13). [104,105]. (14). [114].

#### 4.3.3. Engineering Issues

CW systems are usually constructed outdoors, while multi-stage CWs are usually employed to secure the treatment efficiency. Some engineering issues should be considered in order to correctly use a CW system, as this will enable the maximization of its treatment performance. These include the incorporation of seasonal variety, especially in winters with particularly low temperatures; substrate clogging; wetland plant harvesting; etc. When winter arrives, the low temperature will affect the nitrification and denitrification processes in the wetland, which will, in turn, affect the removal rate of nitrogen from the effluent. In winter, when plants are in a dormant phase, they release less oxygen [115]. Adsorption is a temperature-dependent process: the lower the temperature, the poorer the substrate’s adsorption capacity. Some options, such as the planting of cold-resistant plants, substrate selection, aeration, and the use of insulation measures (covering with insulation film) to produce a greenhouse-like environment, are regarded as effective solutions [115].

The hospital wastewater entering CWs should be pre-treated to avoid substrate clogging [99]. In addition, it should also be clarified whether the toxic substances in the hospital have an impact on CWs. During the pre-treatment period, attention should be paid to the growth of plants, including the prompt removal of growing weeds and the cleaning of broken plant branches and leaves [99]. Although CWs are effective and have low energy consumption, they often necessitate the occupation of a larger area; if this issue regarding the occupied area is ignored, CWs have an advantage over traditional treatments [99]. However, with time, the substrate will become saturated, and the effect of the treatment will be considerably diminished, at which point the substrate will need to be replaced; in this case, the use of a modular wetland will assist in laying and replacing the substrate at the appropriate time [116].

## 5. Hospital Wastewater in the Pandemic Era

The global COVID-19 pandemic remains critical in some countries after almost three years. More than 600 million people across the globe have been diagnosed with COVID-19, and more than 6 million have died as a result [117]. SARS-CoV-2 is highly contagious, particularly with respect to the Omicron BF.7 variant, which is spread primarily through droplets, aerosol particles, and direct contact [118,119].

At present, there are no indications that SARS-CoV-2 present in wastewater is contagious. From February 18 to 2 June 2020, Wu et al. [120] studied SARS-CoV-2 in wastewater from 159 counties in 40 states in the United States; 846 out of 1751 samples were positive for SARS-CoV-2 RNA. SARS-CoV-2 RNA is frequently detected in wastewater [121]. It is unknown whether exposure to SARS-CoV-2 or SARS-CoV-2-RNA containing wastewater is detrimental to humans. Therefore, relevant personnel should follow proper safety measures, such as vaccination and the use of masks and goggles that match the relevant specifications.

The detection of SARS-CoV-2 in wastewater can enable us to infer the presence and severity of an epidemic in a particular hospital or community [122]. Regular detection is necessary to terminate a potential epidemic and avert the resulting harm to human health [123,124,125]. Furthermore, the use of advanced technology (Fenton oxidation) and disinfection-based (ozonation and UV) inactivation of viruses have been proposed in a series of papers as strategies for SARS-CoV-2 control. SARS-CoV-2 is most effectively eliminated from the environment by ultraviolet light with a 254 nm wavelength [126,127,128]. Ozone can denature the lipids and proteins of the SARS-CoV-2 membrane, thus rendering it incapable of infecting humans [49,129]. Zabka et al. [130] utilized ferrate, Fenton oxidation, and related processes to eliminate more than 90% of the SARS-CoV-2 RNA from simulated water.

## 6. Suggestions for Future Studies

Each hospital wastewater treatment process has its own merits, but combinatory processes frequently achieve higher levels of treatment effectiveness. Suggestions for the future directions of studies on hospital wastewater treatment from a macro and micro perspective are as follows:

Macro perspective:

Hospitals generating enormous volumes of wastewater may develop their own wastewater treatment facility/plants, rather than having said wastewater jointly treated with domestic wastewater. This is in consideration of the specific features of hospital wastewater.

The existing literature on the treatment of hospital wastewater by CWs is promising but minimal. Herein, the use of a CW system, specifically, a multi-stage CW, is recommended. Its use is required to assess the treatment capacity of CWs of various sizes for hospital wastewater. The combination of CWs with landscape ecology so as to take full advantage of the landscape value of CWs is highly suggested.

The creation of an integrated treatment system consisting of an MBR combined with advanced treatment processes followed by the use of enhanced CWs would increase efficiency and thus lead to better hospital wastewater purification.

Micro perspective:

The chemical structures of the pollutants in hospital wastewater should be examined in great detail. The specific drugs and chemicals used in the pandemic period and post-pandemic era should be carefully considered to help establish hospital wastewater treatment strategies.

Concerns such as the role of microorganisms in CWs with respect to eliminating pollutants in hospital wastewater; any link between microorganisms and pollutants; and influencing mechanisms and interactions between microbes and plants should be investigated and comprehensively addressed. In addition, the pathways of the viruses surviving and spreading throughout hospitals and wastewater systems and wiser and more effective strategies for the inactivation and disinfection of these viruses should be jointly investigated.

## 7. Conclusions

After examining the characteristics of hospital wastewater and the challenges faced in the pandemic period and post-pandemic era, this paper initially reviewed the application of traditional and advanced treatment processes applied to hospital wastewater. It is reasonable to believe that both the ASP and MBRs offer generally positive performance and should be utilized as key processes for large-scale hospital wastewater treatment. Advanced technologies and processes (Fenton oxidation, electrocoagulation, ultrafiltration, reverse osmosis, etc.) can render hospital wastewater harmless on a small scale, but technical and monetary issues have not been resolved; thus, it is difficult to apply such techniques and technologies to large-scale projects. As a sustainable and eco-friendly treatment approach, in this review, it is shown that CWs have excellent absorption capabilities and can eliminate the majority of pollutants, including viruses, in hospital wastewater contaminants. CWs can be used as a wastewater-sanitizing, post-processing phase after the ASP or coupled with an MBR and some advanced techniques. Certainly, the development a multi-stage CW system is recommended for hospital wastewater treatment, for which various intensifications have been developed so far. The unique ecological and landscape-related value of CWs cannot be obtained through any other treatment technology. Thus, it is reasonable to recommend that an integrated treatment system containing multi-stage CWs is an effective, sustainable solution for hospital wastewater treatment in order to cope with the post-pandemic era. Following the SARS-CoV-2 outbreak and the emergence of the post-pandemic era, treatment technology should integrate the merits of each treatment process, which must be continually optimized and whose essential characteristics must be extracted, thereby leading to the development of an efficient and sustainable treatment approach.

## Figures and Tables

**Table 1:** Recommended antiviral drugs during the epidemic [23].

Name	3D Structure	Formula	CAS Number	Molar Mass (g/mol)
Aciclovir	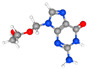	C_8_H_11_N_5_O_3_	59277-89-3	225.2
Zanamivir	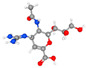	C_12_H_20_N_4_O_7_	139110-80-8	332.31
Amantadine	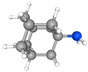	C_10_H_17_N	768-94-5	151.25
Arbidol	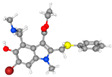	C_22_H_25_BrN_2_O_3_S	131707-25-0	477.4
Amprenavir	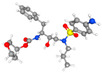	C_25_H_35_N_3_O_6_S	161814-49-9	505.6
Tipranavir	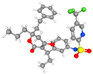	C_31_H_33_F_3_N_2_O_5_S	174484-41-4	602.7
Baloxavir marboxil	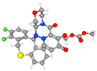	C_27_H_23_F_2_N_3_O_7_S	1985606-14-1	571.6
Tenofovir	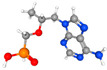	C_9_H_14_N_5_O_4_P	147127-20-6	287.21
Sofosbuvir	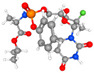	C_22_H_29_FN_3_O_9_P	1190307-88-0	529.5
Darunavir	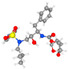	C_27_H_37_N_3_O_7_S	206361-99-1	547.7
Entecavir	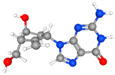	C_12_H_15_N_5_O_3_	142217-69-4	277.28
Ribavirin	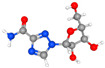	C_8_H_12_N_4_O_5_	36791-04-5	244.2
Remdesivir	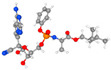	C_27_H_35_N_6_O_8_P	1809249-37-3	602.6
Faldaprevir	NA	C_40_H_49_BrN_6_O_9_S	801283-95-4	869.8
Pleconaril	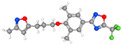	C_18_H_18_F_3_N_3_O_3_	153168-05-9	381.3
Faviparivir	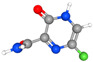	C_5_H_4_FN_3_O_2_	259793-96-9	157.10
Oseltamivir	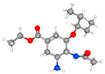	C_16_H_28_N_2_O_4_	196618-13-0	312.4
GS-441524	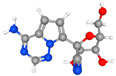	C_12_H_13_N_5_O_4_	1191237-69-0	291.26
Nelfinavir	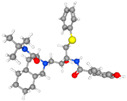	C_32_H_45_N_3_O_4_S	159989-64-7	567.8
Lopinavir	NA	C_37_H_48_N_4_O_5_	192725-17-0	628.8
Indinavir	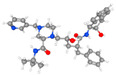	C_36_H_47_N_5_O_4_	150378-17-9	613.8

NA: The structure is too complex without a corresponding 3D structure.

**Table 2:** Traditional treatment, advanced technology, and application of CWs in hospital wastewater treatment.

Number	Country	Details	Treatment	Water Quality Indexes	Reference
1	Spain	The pretreatment employed is coagulation, followed by the activated sludge process and UV/H_2_O_2_ disinfection Volume: 1 L HRT: 48 h SRT: 20–22 d	ASP combined with UV/H_2_O_2_	antibiotics and other drugs	[24]
2	Iran	An aeration tank equipped with an extended aeration device and a submerged biological filter. ASP Type: plug flow Size: L × W × H = 7 × 6.5 × 4 m. Biological filter: Size: L × W × H = 22 × 12 × 1.2 m Substrate: slag (specific surface area 2.49 m^2^/g, porosity 36%,density 2.96 g/cm^3^)	ASP combined with Biological filter	COD, TSS, amoxicillin, ceftriaxone	[25]
3	Vietnam	MBR: Material: glass Size: L × W × H = 0.28 × 0.08 × 0.6 m Membrane modules: hollow fiber Pore size: 0.2 μm Flux: 10/15/20 LMH Ozone reactor: Size: W × H = 8×42 cm Working volume: 2 L Contact time: 20 min Supply rate: (20–40) mgO^3^/h The device runs for: 20 d HRT: 10/6.7/5 h; SRT: 20 d	MBR combined with ozone oxidation	Norfloxacin, ciprofloxacin, ofloxacin, sulfamethoxazole, erythromycin, tetracycline, and trimethoprim	[26]
4	Vietnam	Volume: 8 L Size: L × W × H = 0.28 × 0.08 × 0.6 m Membrane modules: hollow fibers (surface area 0.05 m^2^) Pore size: 0.4 μm Flux: 20 LMH HRT: 8 h; SRT: 20 d	MBR	COD; BOD; ciprofloxacin	[27]
5	Spain	Fe^3+^ source: Fe(NO_3_)_3_, c (Fe^3+^) = 25 mg/L Temperature: 70 °C Initial c (H_2_O_2_) = 2 g/L Initial PH = 3	Fenton oxidation	COD, TOC, drugs	[28]
6	Spain	Temperature: 20 °C Catalyst: c (Fe-BTC) = 0.6 g/L Initial c (H_2_O_2_) = 0.75 g/L Initial PH = 3	Fenton oxidation combined with photochemical catalysis	COD, TOC, drugs	[28]
7	China	The treatment plan: fine grid-ultrafiltration-catalytic wet oxidation Catalyst: carbonized red soil	Catalytic wet oxidation	cephalexin, TOC	[29]
8	Spain	Temperature: 120–150 °C c (catalyst) = 1 g/L	Catalytic wet oxidation	COD, TOC, drugs	[28]
9	Colombia	Material: cylindrical, plastic Size: L × W × H = 20 × 2.7 × 0.3 cm Working volume: 1 L Cathode and aluminum: iron and aluminum, respectively connected to a DC power supply.	Electrocoagulation	COD, BOD, phenols, phosphates, TSS, naproxen	[30]
10	China	Working volume: 120 mL Anode and cathode: IrO_2_/RuO_2_ grids (effective size 2.5 × 2 × 0.1 cm) and graphite felt (effective size 2.5 × 2 × 1.2 cm), respectively, with a spacing of 2 cm.	Electro-peroxone	TOC, COD, NH_3_-N, ciprofloxacin	[31]
11	China	Anode and cathode: platinum plate (3 × 3 cm) and graphite felt (effective area 42 cm^2^) Sacrificial anode: iron electrode (2 × 14 cm) connected to a DC power supply.	Electro-peroxone combined with Sacrificial iron anode	TOC; ciprofloxacin	[6]
12	India	Type: horizontal subsurface flow Material: galvanized sheets Size: L × W × H = 1.2 × 0.6 × 0.6 m Plant: *Australis phragmites* Flow rate: 20 Ld^−1^. The outlet of CWs was connected to a tubesettler	CWs combined with tubesettlers	paracetamol, ibuprofen, carbamazepine, lorazepam, erythromycin, ciprofloxacin, and simvastatin	[32]
13	Saudi Arabia	Size: L × W × H = 1 × 0.7 × 0.6 m Substrate: gravel and sand, Plant: *Phragmites australis* CWs performance was evaluated with respect to pre-monsoon, monsoon, and post-monsoon seasons	CWs combined with tubesettlers	paracetamol, ibuprofen, carbamazepine, lorazepam, ciprofloxacin, sulfamethoxazole, and Fluvastatin.	[33]
14	Saudi Arabia	Material: galvanized sheets Size: L × W × H = 1.5 × 0.65 × 0.5 m Substrate: sand plant: *Phragmites Australis*.	CWs combined with tubesettlers	paracetamol, ketoprofen, carbamazepine, lorazepam, sulfamethoxazole, ciprofloxacin, and Fluvastatin	[7]
15	Thailand	Type: vertical flow Size: L × W × H = 1.5 × 0.6 × 0.6 m Substrate: sand and gravel plant: *Scirpus validus*	CWs	paracetamol	[34]

## Data Availability

Not applicable.
